# Reaction Optimization Experiment for Undergraduate Capstone Organic Chemistry Laboratory Course

**DOI:** 10.1021/acs.jchemed.4c00030

**Published:** 2024-10-08

**Authors:** Jayalakshmi Sridhar, Galina Goloverda

**Affiliations:** Department of Chemistry, Xavier University of Louisiana, 1 Drexel Dr., New Orleans, Louisiana 70122, United States

**Keywords:** Reaction Optimization, Reaction Conditions, Reactant, Reagent, Percent Yield, Purity, Trial Reaction, Modified Reaction

## Abstract

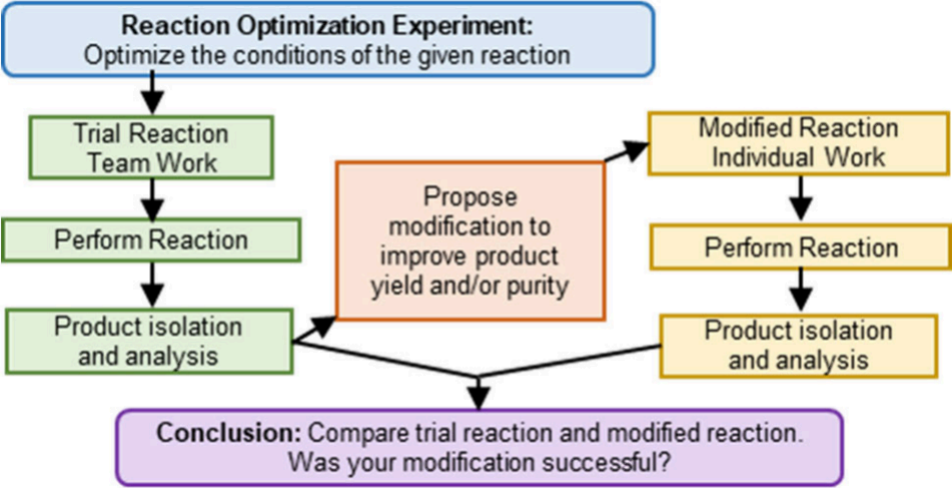

Molecular Structure and Organic Synthesis (MSOS) is an upper-division undergraduate (capstone) laboratory course for undergraduates majoring in chemistry at Xavier University of Louisiana (XULA). The course is designed for juniors and seniors and is based on self-regulated research and learning under limited instructor supervision. It includes a 2-step synthetic project, chosen by each student in the class from a list based on the Organic Synthesis periodical or actual faculty research and then carried out independently. In order to prepare students for their syntheses, we recently included a new project in the course syllabus focused on a reaction optimization that introduces the undergraduate students to the concepts of raising reaction yield, improving product purity, lessening the environmental impact of the reaction, and/or increasing its cost efficiency. A team of 2–3 students performs a preliminary experiment. A rerun by each individual team member incorporating his or her modifications follows this. The goal of this preparatory exercise is to enhance the students’ soft skills, including teamwork, critical analysis of data, and scientific report preparation as well as develop a deeper understanding of the reaction mechanism to make calculated adjustments to reaction conditions for optimization.

## Introduction

Reaction optimization is a broadly defined term that may target a variety of developments in the reaction outcome, ranging from the product yield and purity to economic and environmental improvements. A recent review^1^ identified reaction optimization as being largely unexplored during undergraduate chemistry teaching. This may impede a potential researcher’s skillset in both academic and/or industrial chemical laboratories. Even though so-called “intuition-based” or “one factor at a time” optimization has limited significance in the modern world, training students to think about potential improvements of a chemical reaction is important for stimulating the students’ ownership of laboratory work and enhancing their decision-making skills.^1^ These skills are critical for their future graduate studies as well as for taking up STEM jobs with greater confidence.

A limited number of undergraduate teaching laboratory experiments have been published on optimizing different organic reactions, e.g., the condensation of vanillin and cinnamaldehyde with acetone,^2^ Williamson ether synthesis,^3^ a Grignard reaction between benzyl magnesium chloride and cyclopentanone,^4^ and a Diels–Alder reaction between maleic anhydride and substituted 1,3-butadiene.^5^ Each of these articles is focused on optimizing the reaction conditions for one concrete organic reaction to accelerate the reaction rate or achieve a better yield of the product. Some undergraduate laboratory experiments have incorporated newer techniques of reaction study such as parallel microscale high-throughput study of the Suzuki-Miyaura cross coupling,^6^ multiple reaction monitoring using liquid chromatography-tandem mass spectrometry,^7^ and the integration of green-chemistry practices in reactions toward achieving material efficiency.^8^

**Figure 1:**
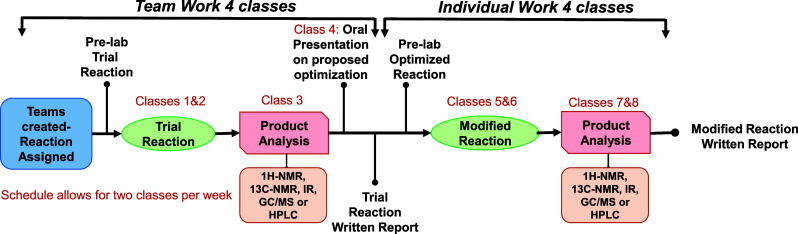
Schematic representation of the components of the reaction optimization project.

The purpose of our approach to teaching organic reaction optimization is more comprehensive, employing widely used chemical transformations performed utilizing traditional processes: in a typical class of 10–12 students, 4 teams of 2–3 students are working on 4 different reactions, and each student “owns” the assigned reaction and comes up with his/her specific modification to improve the product yield, product purity, the economic efficiency, and/or environmental impact of the reaction as detailed in the manuscript (Figure 1). These modifications are discussed within the team and presented to the entire class, which creates an atmosphere of research curiosity and competition between the teams and individual students. A variety of instrumental methods used for the product characterization and results’ evaluation include proton and carbon NMR, IR and GC/MS, or HPLC, which is broader than in the above-mentioned published experiments that use only GC/MS or GC/MS and IR for evaluation. Our experimental design differs from previously reported pedagogical methods for reaction optimization by implementing student training in reaction methods in two distinct stages (Figure 1). In the first stage, students perform the reaction as a team, allowing for deliberation among team members on all aspects of the process. In the second stage, each student performs the same reaction individually with personalized modifications, which enhances their confidence in laboratory techniques.

New innovative pedagogical methods and curriculum enhancement to laboratory courses are initiated and implemented with the goal of improving students’ understanding of practical application of their knowledge, helping them gain proficiency with instrumentation methods and techniques in the laboratory.^9−15^ Course-based undergraduate research experiences (CUREs)^16^ are known to provide STEM research engagement to students with benefits that are similar to undergraduate research internships. The quality and rigor of the experiments in a senior level organic laboratory can provide valuable experience and prepare the students for more independent research skills. These skills can translate to greater success in independent research in graduate schools as well as in professional careers in chemical and pharmaceutical industries. The American Chemical Society Committee on Professional Training (CPT) advocates for 400 h of an in-person laboratory experience in addition to the first year introductory chemistry that can forge the inquiry-driven and open-ended investigations which also promote soft-skills aligned with the view of chemistry as a scientific process of discovery^17,18^ (ACS Guidelines, Paper). In order to address the professional training needs, this paper describes a new experiment in one of our upper-division undergraduate (capstone) courses, “Molecular Structure and Organic Synthesis” (MSOS). The reaction optimization experiment replaced an esterification experiment in the course, wherein the students used a given alcohol and a carboxylic acid to create an ester under catalytic acidic conditions. The esterification experiment was individualized with different alcohols/carboxylic acids and used a standard acid (sulfuric acid) as a catalyst. The new experiment of “reaction optimization” uses a gradual training process for the students to gain confidence in their lab skills.

## History of the Course Objectives

Undergraduate chemistry majors at Xavier University of Louisiana are required to complete a capstone course, which is one of the 4000 level laboratory courses, typically composed of ∼60% seniors and ∼40% juniors. The MSOS course is designed to prepare students for the next step in their career, including graduate/professional school or work in the chemical industry at the technician level. The course objectives are to train the students in organic chemistry laboratory techniques that are routinely used in industrial, academic, and analytical laboratories. The course includes independent projects on qualitative and spectroscopic identification of unknown organic samples and a synthesis project that involves a two-step synthetic sequence of a commercially relevant product. The students are also trained on spectra interpretation, such as IR, proton and carbon NMR, GC/MS, and HPLC spectra.

Over the years of observations, we found that students had difficulty with their transition from performing different qualitative tests and purification procedures on their unknowns to carrying out actual chemical reactions in their synthetic project. In order to fill this gap and prepare students for organic synthesis and their future careers, we recently introduced a new experiment, Reaction Optimization Experiment.

## Goals of the Reaction Optimization Project

The goals of the reaction optimization project were in-depth training for students in nuances of synthetic methodology, reaction process development, literature search, and data analysis along with a concomitant training on soft-skills development including teamwork, oral and written communication, critical thinking, and problem solving. Optimization of the conditions of a given reaction was chosen as the experiment that could be used for achieving these training goals. The project incorporated several activities as illustrated in Figure 1.

**Figure 2:**
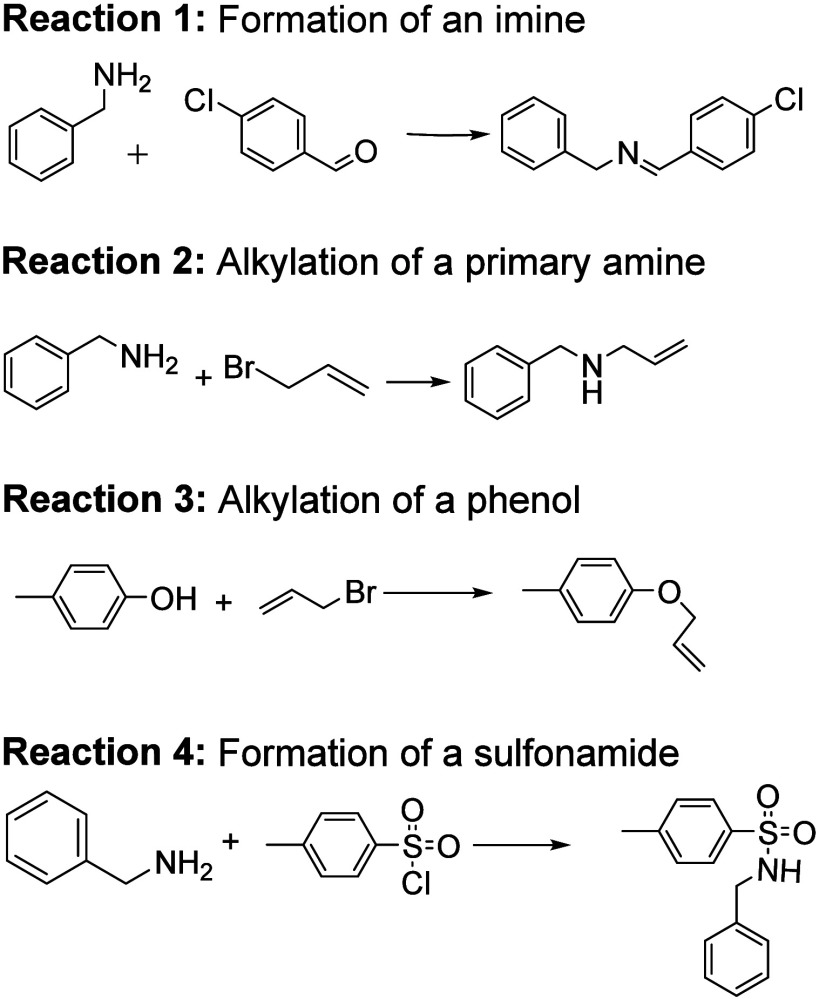
Four reactions chosen for the optimization project.

The project goal is to optimize the conditions of a known reaction. The reactions chosen for this project included four commonly performed reactions in academic research laboratories as well as in manufacturing industries, alkylation of amines and phenols, imine formation, and sulfonamide formation (Figure 2). At a minimum, one of the reactants had aromatic core structures for ease of detection using UV–vis detectors. The students were grouped into teams of 2/3 and allotted one of the reactions. A generic reaction procedure is provided to the students as an initial reference (Supporting Information).

## Description of the Reaction Optimization Project

### Trial Reaction

The ability to coordinate work as a team is an essential skill as most scientific research involves personnel from multiple disciplines. A survey of chemical industries on the skills that are considered essential for bachelor-level graduates are (% of very important responses): strong interpersonal skills (90%), teamwork (83%), and strong work ethic (79%) ranking as more important than problem solving skills (68%), initiative (55%), analytical (44%), and technical skills (35%).^19−21^ Introducing the students to team experiences in the classroom setting will provide them with an opportunity to develop leadership and team skills. Additionally, since this project serves as an introduction to performing an organic reaction, the members of the team benefit from each other’s knowledge and support while performing the reaction. Each team creates an initial prelab report for their allotted reaction. The information in this report includes the reaction scheme, calculations of the quantities of reactants/reagents used in the reaction, reaction procedure reflecting the calculated quantities, theoretical yield calculation, and expected spectra (^1^H NMR, ^13^C NMR, and IR). Feedback is given to the students on any errors in the prelab report. Each team carries out an assigned reaction on a 3 g scale of the limiting reactant, and the tasks are distributed between the team members, including performing the reaction, isolating, and analyzing the product. This part of the project is designed to impart teamwork and team communication skills to the students.

### Analysis of Trial Reaction

Team members obtained all required spectra (^1^H NMR, ^13^C NMR, and IR) for all reactants and reagents used in the reaction and for unpurified reaction products. Quantitative analysis of the reactants, reagents, and the unpurified product was performed using HPLC or GC/MS instruments. Additionally, TLC analysis was performed. The product spectra were analyzed to confirm the structure of the product obtained and to understand the nature of impurities in the crude product, which could be an unreacted starting material and/or reagents used in performing the reaction or isolating the product. Percentage yield of the product is considered one of the measures of success of the reaction performed. The percentage yield is calculated based on the quantitative data obtained through HPLC/GC-MS (detailed in Supporting Information data).

### Trial Reaction Reports

The trial reaction report is communicated by students in two ways: an oral presentation and a written report. The students are trained to use the SciFinder software for a literature search to learn about the types of modifications that have been reported for their reaction. Based on this knowledge and their own understanding, the students are urged to propose and justify modifications in different aspects of the reaction, such as changes to temperature, duration, catalysts, solvents, and reagents used. Each modification should target an improvement of at least one thing: the product yield, purity, environmental impact, and/or cost efficiency. Each team member proposes a different modification. Students are also encouraged to improve on product isolation processes.

### Written Report

A written report consists of several components: (1) a description of the reaction conducted, (2) images and analysis of all spectra and HPLC/GC-MS data, (3) calculation of percentage yield, (4) a preliminary analysis of environmental hazards of all the chemicals in the reaction scheme, and (5) cost calculation of performance of the reaction if it were to be done on a one-kilogram scale of the limiting reactant. The conclusion will include the student’s proposal on modification of the reaction conditions toward their chosen goal.

### Oral Presentation

Students also present the trial reaction results to the entire class as an oral presentation. An oral presentation (time given is 15–20 min per team) is done by each team member presenting the reaction method used, data analysis of the product, and mechanism of the reaction. Each team member outlines their approach toward optimization of the reaction conditions with the goal of improving the outcome of the reaction and the rationale behind it.

There is a question and answer session after each presentation that concludes with the instructor’s feedback and approval of each optimization. A list of modifications proposed by students to the four reactions is given in Tables 1, 2, 3, and 4. The modifications included change in solvents, increase in temperature, changes in stoichiometry, and using a stronger reagent. Several of these proposed changes were based on literature survey of the reaction or the reaction mechanism analysis.

**Table 1:** Modifications Proposed by Students and the Actual Modification Used for Reaction 1: Formation of an Imine

	Student suggested modification	Actual modification used if different from proposed one
1	Change of solvent to methanol based on literature reports	
2	Change solvent to benzene to increase the temperature of reflux	Toluene used as solvent instead of benzene to avoid hazardous chemicals
3	Equimolar stoichiometry instead of slight excess of amine	
4	Use microwave to decrease the reaction time and increase the yield	

**Table 2:** Modifications Proposed by Students for Reaction 2: Alkylation of a Primary Amine^a^

	Student suggested modification
1	Increase the temperature of reaction to 100 °C by changing solvent to dioxane
2	Change of solvent to dimethyl sulfoxide (DMSO), a polar aprotic solvent
3	Change of solvent to ethyl acetate
4	Change of solvent to 2-methyltetrahydrofuran, which is more environmentally friendly and has higher boiling point
5	Utilize a metal catalyst, such as Zinc dust, based on literature reports
6	Increase the excess of allyl bromide since product mixture still contains a substantial amount of benzyl amine
7	Extent the time of the reflux

a Student suggestions used without changes.

**Table 3:** Modifications Proposed by Students for Reaction 3 and the Actual Modification Used: Alkylation of a Phenol

	Student suggested modification	Actual modification used if different from proposed one
1	Change solvent from acetone to acetonitrile, which is more environmentally friendly (based on literature, and it actually worked well to increase the yield)	
2	Use a reagent like potassium	Used sodium methoxide as reagent to enable ease of handling
3	Change solvent from acetone to N,N-dimethylformamide (DMF) (based on literature, and it actually worked well to increase the yield)	
4	Since product mixture has cresol leftovers, increase the excess of allyl bromide	

**Table 4:** Modifications Proposed by Students for Reaction 4: Formation of a Sulfonamide^a^

	Student suggested modification
1	Change solvent to ethyl acetate and added heat
2	Since product mixture has benzylamine leftover, increase the amount of p-toluenesulfonyl chloride to 1.5–2 mol equiv
3	Heat the reaction mixture at reflux
4	Change base from triethylamine to anhydrous pyridine
5	Since adding p-toluenesulfonyl chloride in small portions is associated with bringing more moisture to the reaction, add p-toluenesulfonyl chloride all at once

a Student suggestions were used without changes.

### Modified Reaction

Performance of the modified reaction is the second part of the project that is carried out individually by each student. Students are now rerunning the same reaction on a one-gram scale of the limiting reactant using their experience obtained from a trial reaction. Each student submits a prelab prepared in a similar way as for the trial reaction prelab report, which is reviewed by the course instructor. The review ensures that the required chemicals for the planned modification are available in the laboratory, and the conditions outlined in the procedure are achievable. The reaction scale is reduced to 1 g of the limiting reactant for the modified reactions. This is to ensure that students learn to perform calculations with a change in the scale of the reaction and are able to select glassware fitting a smaller scale. Upon completion of the reaction, students isolate and analyze the product in the same way they did for their trial reaction. The students outline the results of the modified reaction in comparison to the trial reaction and conclude on the success or failure of their specific modification in their final written report. If the modification failed to achieve the goal, then students are encouraged to speculate on a possible reason for the failure.

## Accomplishments and Limitations

Reaction Optimization experiment was implemented for the first time in the Spring 2019 semester. Since then, it has been carried out every semester, except for the Spring of 2020, due to COVID-19 restrictions, for a total of 10 times, and it was carried out by a total of 91 students. It takes 3–4 lab periods to run an assigned reaction in a team and 4 lab periods to run it by an individual student. This time period includes the time spent on running the reaction, product isolation, and the product mixture analysis (collecting all spectra for the starting materials and products). MSOS lab meets twice a week for 3 h, and class size is 10 to 12 students as larger class size would violate the safety considerations.

One of the limitations of the project was the observed differences in participation among the students on the team. This lack of participation becomes more evident when students work individually in the second part of the Reaction Optimization project, where each student collects and interprets his or her own spectra. It is also reflected in the modified experiment reports, which represent individual work rather than collaborative team efforts, as in the trial reaction. Another limitation is that not all four offered reactions are equally challenging in terms of execution and NMR spectra interpretation. The instructors are continually seeking new reactions to offer. Designated class times were utilized for performing the reactions and product analysis using spectroscopy. SciFinder search, analysis of results obtained, prelabs, and preparation of written and oral reports were accomplished by the students away from class.

## Assessment and Evidence for Positive Learning Outcomes

Evaluation of the students’ learning is conducted by several means: two written prelab reports, two written lab reports, oral report on modifications for the assigned reaction, calculation/scaling questions on a midterm exam, and a survey questioning their preparation for the synthetic project, which is the main project of the course. For most students, the grade for the modified (individual) reaction prelab is usually 28–39% higher than for a trial (team) reaction; the grade for the modified reaction (individual) reports is usually 7–21% higher than trial reaction (team) reports. Specifically, the ability of students to scale the reaction, write a scaled procedure, and predict spectra in the prelab and describe observations, interpret the actual IR, NMR, and GC/MS or HPLC spectra and the logic of description were main constituents on the grading scale.

In this course, the reaction optimization project is followed by a synthesis project. Students are offered an assortment of possible multistep synthetic projects to choose from. Most of the procedures are taken from the Synthesis periodic, and some are based on faculty research projects. The synthesis project involves the students performing a 2-step synthesis of a molecule that has commercial utility. According to a survey, conducted at the end of semester to examine the impact of the Reaction Optimization project, the students felt highly confident in their abilities of performing the synthesis project, which involved calculations for the reactions, performing the reactions, and isolating and analyzing the products (Supporting Information Tables S1 and S2). These were skills that they had encountered in the Reaction Optimization project. The grades for the synthesis steps prelabs and lab reports submitted by students after accomplishing the Reaction Optimization project were about 35–40% higher, as compared to the corresponding grades of an average student before the implementation of this project. Based on the survey free response section, students indicated that they would welcome more group work and more hands-on guided experience, and some even called for bigger changes to an assigned reaction, as they felt like they did not make major changes to the reaction process. We think this may be indicative of the students’ interest toward research, which could be an indirect proof of the Reaction Optimization project success. Most of the other suggestions were about giving more time for different project activities, specifically about allotting some class time for searching SciFinder and brainstorming possible reaction modifications within the teams. We plan to introduce these suggestions in the next semesters.

## Hazards and Safety Precautions

Before being engaged in a reaction optimization experiment, students are sufficiently trained on safety precautions: they are required to give all information about each chemical used in the table, including the hazards associated with each, and they can start a reaction only after their prelab is approved. In addition, demonstrations of a special safety session are conducted before the experiment that illustrate how things can go wrong in an organic lab, how to work safely when handling hazardous chemicals, and types of immediate actions that can be taken in case of an accident. No chemicals used in this experiment have bio-, radiation-related, or other special hazards, and they are used in relatively small amounts. This experiment was conducted by 10 different groups of students (91 students total), and no hazardous situations occurred.

## Conclusion

Overall, we think that implementation of the Reaction Optimization project was successful and helpful in the enhancement of the students’ soft skills, including teamwork, critical analysis of data, and scientific report preparation, as well as in the development of a deeper understanding of the reaction procedure and mechanism. This conclusion is drawn based on 10 semesters of running the experiment by students and comparing their ending preparation and skills to multiple groups of students that did not encounter the Reaction Optimization project.

As compared to students taking MSOS before Fall 2020, students from the last 10 semesters demonstrated a better understanding of a reaction procedure and an increased confidence in carrying out their individual synthesis projects. Most students became proficient in scaling reactions, planning their reaction setups, and understanding the purpose of each reagent and action, which was not the case before Fall 2020. They also required less assistance in assembling the glassware and equipment, as well as in running their individual synthesis steps.

These observations/conclusions agree well with students’ survey responses from a total of 33 students (Supporting Information Tables S1 and S2). Specifically, the fact that all 33 students responded that the experiment helped to improve their laboratory and critical thinking skills, of whom 20 strongly agreed is indicative of success in achieving the purpose of its implementation.

## Supporting Information Available

Course syllabus, survey results, student handout for the reaction optimization experiment, grading rubric for oral presentation (PDF, DOCX)
