# Supplementary material for: Reaction Optimization Experiment for Undergraduate Capstone Organic Chemistry Laboratory Course
Source: J Chem Educ. 2024 Oct 8;101(11):4680–5. doi: 10.1021/acs.jchemed.4c00030 (PMC11562579; doi:10.1021/acs.jchemed.4c00030)
Supplement: Supplementary file 1 — ed4c00030_si_001.pdf [file ed4c00030_si_001.pdf]

Supporting Information:

Reaction Optimization Experiment for Undergraduate Capstone Organic Laboratory Course

Jayalakshmi Sridhar* and Galina Goloverda

Department of Chemistry, Xavier University of Louisiana, 1, Drexel Dr., New Orleans, LA 70122.

* Corresponding author Ph: 504-520-7519, Email: jsridhar@xula.edu

Table of Contents

Description	Page
Syllabus of the CHEM 4320L course	S3
Student handout for the reaction optimization experiment	S8
Student survey	S15
Student survey results	S17
Grading Rubric for Oral Presentation	S20

Molecular Structure and Organic Synthesis Laboratory Syllabus CHEM 4320LB Spring 2024
2 Semester Hours
NCF Annex, Room 360

Section 01: Monday and Wednesday 2:00 – 4:50 PM **Instructor Name**

Office:
Office hours:

Course Description: Identification of unknown materials using qualitative and spectroscopic methods is explored. A variety of chemical purification and spectroscopic characterization methods are used. Optimization of reaction conditions related to process chemistry is explored. The economic and environmental impacts of synthetic routes are analyzed. Students perform multi-step synthesis followed by isolation, purification and characterization of products. The students are introduced to Good Laboratory Practices and Scientific Search tools. This course seeks to bridge the gap between the elementary organic lab and the advanced organic methods practiced in research and development division of pharmaceutical and chemical manufacturing industries. It allows the student to develop critical reasoning skills, computational skills, team work, **self-regulated learning**, and communication skills (oral and written presentation) that are required for a professional career.
As an upper level course **this class does not reteach** the material from the prerequisites. IR/NMR spectroscopy lecture is the only one that we offer as a way of a review. Students must review all the theoretical and practical organic chemistry concepts and skills from CHEM 2210/2220/2230L/2240L, and contact the instructor individually during office hours **ahead of each class** if they need help.

Prerequisites: C or better in Chem 2220 Lecture/Drill, Chem 2230-2240 Labs (Organic Chemistry)

Expectations: knowledge of the theory & elementary techniques learned in Organic I and II labs, such as acid-base reactions, recrystallization, TLC, extraction, distillation, etc.; stoichiometric calculations, including determining a limiting reagent, theoretical and percent yield; ability to interpret IR, proton and C-13 spectra. You will also be expected to prepare stock solutions and qualitative test reagents from solids or more concentrated commercial solutions.

Required Text (and other required material):
1. Zubrick, J. W. *The Organic Chem Lab Survival Manual*, 7th ed., John Wiley & Sons, Inc., New York, 2008. (previous editions are acceptable)
2. **Bound** notebook for the laboratory (Your lab notebook from Quant can be used **but only if there is enough consecutive pages left for this class**, ~40 pages.)
4. Lab coat, goggles, closed-toe shoes and calculator

Additional books suggested for reference, available in the library and/or in the lab:
• Shriner, R. L.; Hermann, C.K.F.; Morrill, T.C.; Curtin, D.Y. ; Fuson, R. C. *The Systematic Identification of Organic Compounds*, 7th Ed.; J. Wiley & Sons, Inc. New York, 2004.
• Williamson, K. L. *Macroscale and Microscale Organic Experiments*, 4th ed., Houghton Mifflin Company, New York, 2003.
• Silverstein, R. M.; Webster, F. X. *Spectrometric Identification of Organic Compounds*, 6th ed., John Wiley and Sons, New York, 1998. (Copies are in lab and on reserve)
• Baron, N. *Escape from the Ivory Tower;* Island Press, 2010.

<u>In the event of the University closure,</u> assignments and other course materials will be posted on Brightspace. Students will be informed of the class expectations by e-mail, the Xavier Chemistry website and/or the Brightspace website. It is the student's responsibility to check the websites and his/her Xavier e-mail account for information.

Course Objectives and Student Learning Outcomes:

• To develop qualitative thinking skills and problem solving techniques through the identification of unknowns and data analysis. These skills are needed in any scientific or technical career.

• To develop the ability to organize and carry out a scientific investigation independently.

• To develop the laboratory skills and techniques (both instrumental and chemical) used to identify and characterize organic compounds, both unknown and products of reactions.

• To develop the laboratory skills for reaction conditions optimization for large scale processing.

• To develop the skills and technique to carry out a multi-step synthesis.

• To develop and strengthen the skills to present laboratory results both in writing and orally.

• To develop and strengthen library research skills and good laboratory practices.

Course Requirements: Students are expected <u>to be prepared for</u> and attend every class meeting as scheduled; there are no make-up lab sessions. **Students who <u>miss 3 or more classes</u> cannot pass the course.** Students are also expected to check the Brightspace and Xavier e-mail account for information regularly. The midterm exam is open book and will cover information related to the identification of unknown compounds, including spectroscopy and optimization of reaction conditions. The *Lab Performance* grade includes factors such as: preparation for each class ahead of time, initiative/responsibility, attitude towards learning, effort, following directions, being on time for class, wearing safety goggles, cleaning up the work space and *<u>NOT using cell phones, including earphones</u>*. **Of the 75 points, one point per day will be awarded for daily check-out. It is the student's responsibility to find the instructor for a check-out**.

Course Assignments and Evaluation: *Introductory take-home Quiz 10 points*
Unknown #1 Report (preliminary report = 30 points and Unknown identification report = 70 points; Total = 100 points)
Unknown #2 Report (35 points)
Quiz on basic organic techniques (TLC, Reaction calculations and Recrystallization) (25 Points)
Squalor Exercise (20 points)
Reaction Optimization Experiment – Prelab for a Trial Reaction (25 points)
Reaction Optimization Experiment – aTrial reaction report (50 points)
Reaction Optimization Experiment – Oral Presentation of the Reaction Optimization Strategy (25 points)
Reaction Optimization Experiment – Prelab for a Modified Reaction (25 points)
Reaction Condition Optimization – Modified reaction report (50 points)
Spectroscopy problem sets 4 in number (25 + 25 + 25 +25 = 100 points)
Midterm Examination (100 points)
Step 1 and Step 2 Synthesis pre-labs (25 points x 2 steps = 50 points)
Report for the step 1 and step 2 of syntheses (50 points x 2 steps of syntheses = 100 points)
Final Report on synthesis (50 points)
Oral Report (25 points)
Notebook (45 points)
Lab Performance (75 points)
Total Possible = 910 points

The method of assigning letter grades will not be more stringent than the following:
A, 90% and above; B, 80-89%; C, 70-79%; D, 60-69%; and F, below 60%.

Assignments are due at the beginning of the class unless otherwise noted. **No late problem sets will be accepted**. If one of the other assignments is submitted late, **10%** of the points for that exercise, ***per day*** (not per class) will be deducted. Assignments turned in too late to receive a credit must be still turned in to pass the course, no exceptions. Final Report on synthesis project is considered as a final exam for this class, and it will not be accepted late. Any other missing assignments will not be accepted after the Quiet Day. All students must be present for each oral report and participate actively.

Academic Integrity: The following is quoted from the Xavier University Faculty Handbook:
"If a student's test, examination paper, laboratory report, term paper, or other written assignment gives evidence of not being completely his/her own work, he/she may be given an F for the course. A student who communicates with anyone during the course of an examination or test, unless with the permission of the instructor, may be immediately dismissed from the room and given an F. Such communication includes attempt to read from another's paper. If a student is found to have brought study materials into the examination room without the instructor's permission, it may be assumed that he/she intended to use such materials unlawfully, and he/she may be penalized accordingly." Students should also consult the Academic Integrity Policy:
http://www.xula.edu/cas/documents/cas_academicIntegrity.pdf

Laboratory Notebooks:
Lab notebooks are used by scientists to record data *as they are collected and not later*. Another person reading the notebook should be able to tell what experiments were done, what results were obtained, and when. Important questions involving priority, patent rights, and scientific fraud are often settled by referring to original data in lab notebooks. Therefore, it is important that they be kept correctly.
You must use a bound notebook and write **in pen**. You may use the notebook you have from Quant Lab, **if enough consecutive pages are remaining**. Loose-leaf and spiral notebooks and (especially) scraps of paper are not permitted. Cross out mistakes once; white-out may not be used. Pages may not be torn out. Write only the results of your lab work; do not include a pre-lab lecture notes. Clarity and sound organization are prime virtues in a lab notebook. Neatness is nice, but it is often impossible. However, you must write legibly so that others can read it. The notebook should be dated and signed each day you work. The first page of your notebook must be a Table of Contents. All pages must be numbered. **You are required to write at least 3 planned specific objectives in your notebook ahead of each class.** These will be graded as part of the notebook grade. Start each new unknown or new synthetic procedure on a new page. For the unknowns, your data should include the measurements obtained (such as BP/MP) and not just the tests performed but also your observations, ie, green ppt formed or a color changed. Include other information such as what spectra you obtained. Be sure to give the identity of the unknown at the end. For the reactions, you need to include the pre-lab write-up and any changes made to this procedure when you ran the reaction. Observations and spectra obtained are also included. Notebooks will be evaluated at the end of the semester. Your instructor may inspect your notebook at any time during the semester, without warning.

Unknown Reports:
The **first** unknown has a **preliminary report** (30 pts) and a **final report** (70 pts; word processed). For a preliminary report, the results of a specific battery of tests are tabulated on a form, and you will learn if you are on the right track in determining the unknown. The final report is a discussion of how a student determined the identity of the unknown and why other compounds are not the answer. This discussion must describe your thought process thoroughly, for a maximum credit. Logic of your description will be part of your grade. I need to know how you arrived at your decision and what was most helpful.
The **second** unknown does not have preliminary report, it will be identified using qualitative tests and at least three out of 4 spectroscopic methods (IR, ^{1}H NMR, ^{13}C NMR, GC/MS) (35 pts)

Reaction Condition Optimization (RO): A specific reaction is assigned as the reaction to be optimized (see additional handout for details). This will be a team project (team of 3 students). Reaction will be performed in two different ways and include one/two steps. The stoichiometry of the reagents, solvents and drying agents, time and temperature of the reaction may be varied. The products will be isolated, purified, and a percent yield will be calculated. Pre-labs will be submitted before performing the reactions. Two reports are generated outlining and comparing the results from the two experiments. The reports will include a cost analysis and an environmental impact analysis of all of the reactants and reagents involved to understand the economic and environmental impact. An oral presentation detailing the reaction condition optimization strategies will be given in teams.

Reports on a Synthetic Project (SP):
Each of the synthetic steps has two written assignments, and in addition there is a final report and an oral report. For the two steps of the syntheses, a detailed pre-lab write-up must be turned in and approved before a student can begin that procedure; see a handout for details. After each synthetic reaction is completed, you will write out a complete experimental procedure, including the characterization data, in a *Journal of Organic Chemistry* experimental section's format (www.chemistry.org; choose publications tab; find JOC). You will need to discuss whether the reaction worked and how you knew. Product obtained in each step is purified and reanalyzed. The final, summary report is modeled on a *JOC* Note. Consult recent issues of *JOC* in the pharmacy library or online for examples, and handouts for more specific information. An oral report is a 15-minute presentation summarizing the final written report on your synthetic project.

Illness Contingency Plan: Please do not come to class or to my office if you have symptoms suggestive of a contagious disease (e.g., significant fever, a recent diagnosis of a contagious disease, sneezing or cough not associated with allergies). Please notify me as soon as possible if you know that you will miss class(es) due to illness. Students who miss class due to illness will be able to access course materials online in Brightspace, and I will work with them individually.

If you are required to enter quarantine or self-isolation for any reason, please contact Student Health Services first who will then contact your academic Dean. Professors will be formally notified that you will miss class. You are not required to provide confidential health information, but you may be asked for a proof of seeing a health professional (e.g., return to class documentation). As part of our commitment to maintain confidentiality, to encourage more appropriate use of healthcare resources, and to support meaningful dialogue between instructors and students, the Dean's office will only share that a student has a legitimate excuse for missing classes.

Other Comments:

1. You are more like an independent researcher in this class with all the responsibilities of making the informed decisions where appropriate. It is assumed that you know all previously learned techniques from Organic I and II labs. Planning each day of your lab work is part of the responsibilities.

2. If you encounter a problem or are not sure what you're doing, ASK.

3. My answer to a question "Is this a positive test" is "I don't know". If you want to see what a positive (or negative) test looks like, find a compound you know will give the appropriate result, and test it. Compare these results for the known compounds with the results from your unknown.

4. Cell phones, and other electronic communication devices **must be turned off** during class.

5. No eating, drinking or horseplay in the lab. Safety goggles **must be worn over the eyes** at all times in the lab, and sensible clothing (including closed-toe shoes and no Crocs) must be worn. **Not wearing goggles, coming late to class, wasting the class time and using cell phones, including ear phones during the class will cost you performance points, at your instructor's discretion.**

6. **In the lab, spend time on things that can only be done in lab; plan and prepare before coming to class! Do not wait until the last moment with questions about the assignment or lab project.**

7. <u>Your positive attitude, effort & progress are among the most important things for this class</u>.

Student Handout for the Reaction Optimization Experiment

A reaction optimization experiment will be performed by students in teams of 2-3/team. Each team will be assigned one of the following reactions. The students have to complete an initial prelab, perform the reaction (3 g of a limiting reactant scale – this will be called a '*trial reaction*') as a team, and analyze the obtained product (1H-NMR, 13C-NMR, IR, GC/MS or HPLC). At the same time, the team also has to obtain information such as a mechanism of the reaction, alternative methods for the same reaction (using SciFinder), a cost + an environmental impact of all of the reactants/products/reagents of the assigned reaction (these tasks can be divided up between the team members). Upon completion of the reaction and product analysis, the team has to come up with 3 ways to modify the reaction to improve the yield or purity of the product or other things like cost of the materials, environmental impact. Modifications can include a change in the molar ratio of the reactants/reagents, the types of reagents or solvents, the reaction temperature, the reaction time and/or a change of reagents/solvents to a more environment friendly ones. Each team member will then re-run the reaction at a 1 g of a limiting reactant scale using a different modification, which will be called a '*modified reaction'*. The product obtained from a modified reaction will be analyzed by 1H-NMR, C-13 NMR, GC/MS or HPLC and IR. The final conclusion will be drawn by each student as to whether their modification was successful in improving the reaction in terms of the product yield or making it more cost/environment friendly.

Reaction 1:

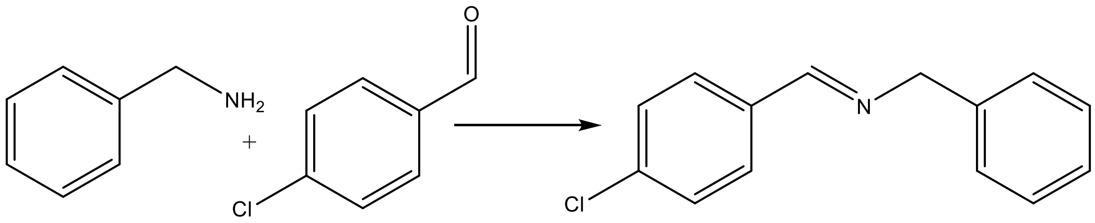

The imines were prepared in quantitative yields by simply refluxing the aromatic aldehydes (0.1 mol), the amines (0.11 mol), and an anhydrous $MgSO_4$ (0.15 mol) in dichloromethane (25 ml) for 2 h, followed by filtration of the reaction mixture through a pad of Celite and subsequent removal of dichloromethane from the mother liquor.

Reaction 2:

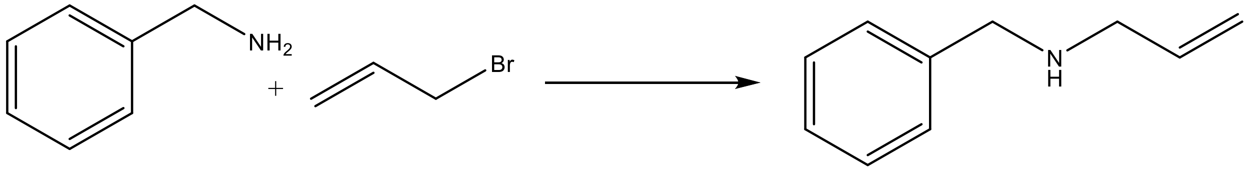

An unhydrous tetrahydrofuran (20 ml) was added to a mixture of benzylamine (9 mmol), allyl bromide (9.9 mmol) and an anhydrous potassium carbonate (11 mmol) (K_2CO_3, potassium

carbonate should be dried by heating in the oven in a beaker for 24 h) under nitrogen. The reaction mixture was heated at reflux for 1hr. The reaction mixture was then cooled to room temperature and vacuum filtered to remove an unreacted potassium carbonate. Solvent was then evaporated under reduced pressure to give the product as yellow oil.

Reaction 3:

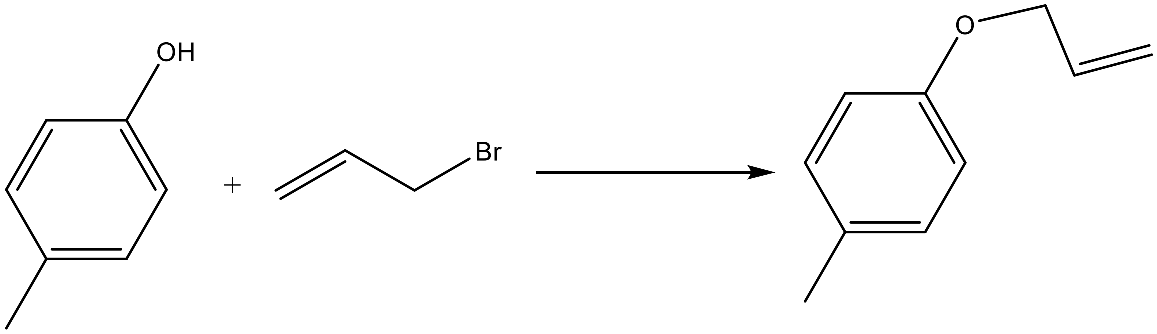

A mixture of 0.2 mole of *p*-cresol, 0.23 moles of allyl bromide, 0.2 mole of an anhydrous K_2CO_3 (potassium carbonate should be dried by heating in the oven in a beaker for 24 h) and acetone (200 ml) was stirred for 24 hours. Then 150 ml water was added and the mixture was extracted with ether twice. The combined ether solutions were washed with 10% NaOH solution and then with brine. The ether solution was dried over Na_2SO_4. Upon concentration the product was obtained.

Reaction 4:

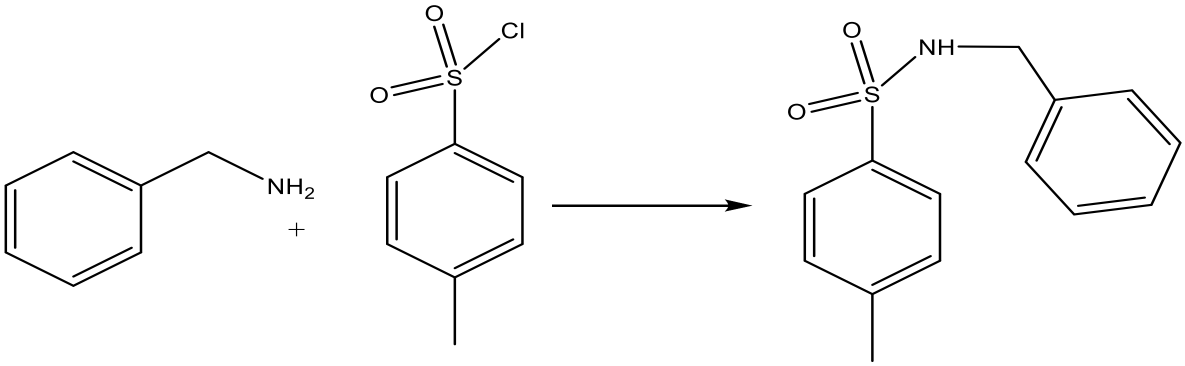

To a mixture of benzylamine (20 mmol) and triethylamine (30 mmol) in 30 mL of dichloromethane was added p-toluenesulfonyl chloride (22 mmol) in small portions, the reaction mixture was then stirred at room temperature for 1 h. The mixture was quenched with 50 ml of water; the aqueous layer was extracted with dichloromethane (3 x 20 mL). The combined organic layers were dried over Na_2SO_4, and solvents were evaporated under reduced pressure to give the product.

Product Analysis:

First, you need to determine the **crude yield** of the product, which is most of times a mixture of the targeted product, unreacted starting material(s), side products and other impurities. If your isolated product is a solid, try to collect as much of it as you can, make sure it is dry and weigh it. If your product is an oil, it might be hard to collect all of it, so weigh the flask with the oil and analyze the product. You will need to weigh that same flask again when it is empty and dry. The difference in mass will be your isolated yield.

You will be using spectra such as ^{1}HNMR, ^{13}C NMR, IR and GC/MS or HPLC (three spectra out of four will be ok) to analyze your reaction outcome.

^{1}H NMR, ^{13}C NMR, IR and GC/MS (or HPLC) spectra will need to be taken for your reactants as well as the product obtained to compare and analyze. GC/MS or HPLC will provide you with % purity of your product. You may also run a TLC plate of the product versus starting materials. There could be an unreacted starting material in your product mixture.

Calculation of Yield:

Your prelab will include a theoretical yield calculation.

After performing the reaction, the mass of the product isolated will give you the reaction yield. From GC/MS or HPLC you will determine the purity of your isolated reaction product (we will call it an isolated product), which can be used to calculate the actual % yield of the targeted product.

For example:

Your theoretical yield for the reaction is 2.3 g.

The isolated crude product weighed 2.7 g.

GC/MS or HPLC indicates that only 65 % of the isolated product mixture is your actual product. Then 65 % of 2.7 g is your actual product yield, which comes to 1.76 g.

So, your % yield = 1.76 g/2.3 g X 100 % = 76 %.   Or 2.7 g/2.3 g x 0.65 x 100 % = 76 %

Reports for this experiment (they must be typed):

Prelab for a Trial Reaction (25 pts.):

Calculations for your trial reaction will be based on 3 g of your limiting reactant. So, the first step for you would be to analyze the reaction + procedure given to you and identify the limiting reactant. Use the stoichiometry given for your procedure to calculate the quantities of the second reactant and all other reagents. Use appropriate size glassware.

Your prelab must be typed, except for a reaction scheme and calculations, which may be legibly handwritten. Organize your prelab using exactly the same subtitles as specified below and in the same order.

Your prelab should include:

Title of experiment (tailored to your example, not the one out of the book)

Literature citation(s): Use SciFinder to find the literature citation for your reaction.

Reaction scheme (ie structural drawing of the reaction using your molecules, get the names of the reactants and products using ChemDraw)

Table of reagents: include as much information as possible on the chemicals you will be using. Your table should include: chemical name, structural formula, molecular formula, molecular weight, BP or MP, density for liquids, solubility if known, cautions/hazards. Most of this information is available in a chemical catalog (Aldrich, Acros) or in the CRC. You may not have full entries for all of your compounds, but do the best you can.

Calculations: clearly show how you calculated the amounts for each chemical, and show the theoretical yield calculations. If the amounts are incorrect but I can see where your mistake is from the calculations shown, you will receive a partial credit.

Scaled procedure it should reflect what and how exactly you will run the reaction; it should contain your calculated amounts of all chemicals, conditions, glassware you will use, and it should include a theoretical yield. Use a paragraph format rather than a cookbook list.

As you work on this exercise, be sure to keep an accurate notebook.

Product Analysis:

You will be obtaining spectra such as ^{1}H NMR, ^{13}C NMR, IR and GC/MS (or HPLC) to analyze your product. Your prelab should include prediction of ^{1}H NMR, ^{13}C NMR and IR spectra. Use format given below for ethyl benzoate for your predicted spectra: show ^{1}H NMR and number carbons for ^{13}C NMR on the product structure directly and specify the appropriate bonds for IR peaks.

Report on a Trial Reaction (50 pts.):

Start with describing your observations during the reaction, e.g.: reactants (name them and give the amounts and moles for each) readily dissolved in solvent (name it and give the volume you used) forming a clear colorless solution; after 10 minutes of refluxing the color changed to a pale yellow, and it deepened towards the end of the reaction; a thick white precipitate formed on the 20th minute of reflux or gas was evolved, etc. Think of this as an executive summary, rather than a repeat of your notebook. Comment (1-2 sentences) on stoichiometry of reactants/reagents and the time of the reaction (NO tables of reactant/reagents). Describe any problems you encountered and deviations from the original procedure. You will also need to comment on the product obtained, describe its appearance and report isolated % yield. You would have taken the spectra for the reactants and the product obtained. First, think about the changes in the spectra that you would ***expect to see*** if your reaction worked. Then compare the spectra of the product obtained with what you expected to see. In some cases you can look this up, and in ALL cases you can just think it through.

EXAMPLE: if the expected product is ethyl benzoate.

IR (cm^{-1}): 3050 (sp^{2}CH) , 2950(sp^{3}CH), 1750 (C=O), 1650 (C=C)

Draw the structure of the expected product and assign the ^{1}H NMR signals to specific Hs as shown in this picture. Also number all carbons that will show separate peaks in ^{13}C NMR and predict their chemical shifts to each carbon (it is not always possible to predict the exact numbers, but do your best).

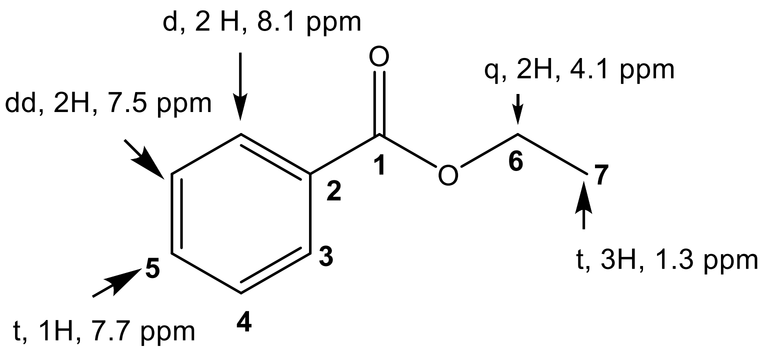

^{13}C NMR (ppm): 175 (1), 135 (2), 133 (5), 130 (3), 126 (4), 75 (6), 20 (7)

Then discuss the identity and purity of the product, and include a full analysis of the spectra you actually did obtain. You are encouraged to show structure and peak assignment directly on each spectrum, but you should also interpret/discuss each spectrum in your report: use a separate paragraph for discussing each spectrum. Comparisons to the starting materials are expected. There will be differences, but also a large number of similarities. **In addition, if your spectra have signals that do NOT belong to your compound or reactants, you need to identify them as that of the solvents/reagents as best as you can.** For example, you would need to identify a solvent that had not been removed; an unreacted starting material; known or unknown byproducts. At a minimum (last resort), indicate an "unknown impurity". Importantly, please keep in mind: if it is obvious to me that particular peaks in your spectrum are from the solvent that you used, but you don't recognize this, some points will be lost.

Product Purity:

GC/MS: using integrated chromatogram and mass-specs of particular peaks determine the composition of the product mixture. From GC/MS results you should report % of the actual product and % of each other identifiable component of the product mixture that you isolated. You might not be able to identify each peak in the chromatogram, but do your best. Think of your starting materials and possible byproducts. Remember that you already have done GC/MS for the reactants.

HPLC: % reactant A, % reactant B and % product. HPLC results should be described clearly as to the % of the product mixture that you actually obtained. Try to figure out what the impurities could be. Remember that you already have done HPLC for the reactants. So, you can compare their retention times (*Rt*) with that of your product and whether it matches with the *Rt* of the authentic product (it will be given to you as a solution).

Include in your report: a scheme of your specific reaction; a percent yield calculation, literature citation(s), cost and environment analysis of your reaction (see a guide to this below), and a curved-arrow mechanism for your reaction. This mechanism is elementary, and it can be found in any organic textbook. Make sure to draw the arrows correctly!

Economic (cost) and Environmental Impact of the Reactions: Upon completion of a trial reaction and a modified reaction as part of the reaction optimization experiment, you have to analyze the impact of your reaction modification on the cost and the environmental effects of the original reaction. Did your modifications increase or decrease the total cost? Is your reaction more environmentally friendly after modification? This analysis should be part of a trial reaction report and also a modified reaction report.

The report on cost of performing the reaction should consist of the following:

Draw the reaction scheme. Then draw a table to include all of the reactants/reagents and the amounts of each chemical used in the reaction, based on 1 kg of a limiting reagent. Chemicals are usually sold in particular amounts per pack/bottle. Make sure to include the price per unit and a total cost for each chemical. E.g. according to your calculations you need 1 L of a solvent, which is sold in bottles of 100 mL for $43 each. So your total will be $430.

- The cost of a trial reaction/modified reaction if a limiting reagent's quantity were 1 Kg. (Remember that you have to scale-up all other reactants/reagents before calculating the total cost. You can get a price for the chemicals from the catalogs available in the lab).
- Include a labor cost at $100/hour, but don't count time while your reaction is left to stir until the next class: only include the time when you were actually working.
- Include a heating cost if applicable at $50/hour.
- Analysis should include your thoughts on the results of the modified reaction in terms of % yield of product and whether the increase in cost (if applicable) was justified.

An environmental impact should include the effect of all of the chemicals used and produced in the reaction, and the best way to dispose of all of the unwanted products. Think about reusing any of the excess reagents/reactants, which would make the reaction more efficient.

Oral Report (25 pts):
An oral report will be scheduled after completion of a trial reaction and before the prelab is due on a modified reaction. The oral report will be short presentations by each member of the group on the mechanism of the reaction, other synthetic methods for the same reaction identified through a Scifinder search and an impact on the environment of the reagents/products. Each of the students will also discuss their proposed reaction modification with clear explanations of why they are planning that particular modification. After the in-class discussion, each student must send a brief email to the instructor, summarizing the proposed modification before the end of the day of the oral report. Part of the grade for the oral report is reserved for this email.

Prelab for a Modified Reaction (25 pts): This prelab should include-

Title of experiment: (tailored to your example, not the one out of the book)

Literature citation(s): Use SciFinder to find the literature citation for your reaction.

Reaction scheme: (ie structural drawing of the reaction using your molecules)

Modification planned and the Thought behind the Modification: Give an explanation of why you are doing the modification that you have planned for the assigned reaction. It could be a reason such as the reaction mechanism suggests this reagent can have positive effect due to its role in ….. or temperature increase can increase the rate of the reaction…., longer reaction time can ensure completion of the reaction….., this solvent is more environmentally friendly as the original solvent had this harmful effect…..,etc. If your modification is based on a literature reference, you must discuss the source's justification AND print and attach to your pre-lab this article or a particular page from the referenced article, if the article is too long, and highlight the relevant part. If you print just one page, make sure it has full reference to the article, e.g. "The article's title", *J. Org. Chem.* 2018, 83, 14102−14109. Use this same style for all of your literature references, giving a web link only is insufficient.

Table of reagents: include as much information as possible on the chemicals you will be using. Your table should include: chemical name, structural formula, molecular formula, molecular weight, BP or MP, density for liquids, solubility if known, cautions/hazards. Most of this information is available in a chemical catalog (Aldrich, Acros) or in the CRC. You may not have full entries for all your compounds, but do the best you can.

Calculations: clearly show how you calculated the amounts for each chemical, and show the theoretical yield calculations.

Scaled procedure use the same format as for a trial rxn & include theoretical yield.

Report on a Modified Reaction (50 pts): This report will be very similar to that of the trial reaction report. In addition, write a summary to state explicitly if the reaction modification worked out the way you predicted it or not, and if it worked, how well. It is perfectly possible that the GC/MS looks terrific, but the proton NMR shows just a solvent; be prepared that not all of your spectra may lead you to the same conclusion. Also describe things that might have gone wrong and/or things you would do differently if you did this reaction again. Include comments on things that were important that you did do correctly, eg, "The acid was added dropwise, which was important, because the solution built up heat."

As the semester proceeds, the quality of your spectra will count more and more in your grade. NMR spectra should have good signal to noise ratios, though we do understand that not all of the carbons are easily seen in ^{13}C spectra (generally, it can be difficult to observe a C without an H). Proton NMR spectra should be well phased, referenced correctly, and should have integrals for all-important signals.

Remember to include a cost & environmental analysis for your modified reaction.

MSOS Student Survey: Learning Outcomes of the Reaction Optimization Experiment

The purpose of this short assessment is to gain insight into your experiences of the Reaction Optimization Assignment. Your feedback is extremely useful. Completion of this assessment should take no more than five minutes, is anonymous (that is, you do not have to provide any identifying information), and voluntary. You will have the option to leave comments if you wish.

1. Did you know about the SciFinder tool before this class? (Please circle one answer)

YES **NO**

2. Did your reaction optimization work in terms of: (please circle one answer to each statement)

Increasing the product yield **YES** **NO**

Improving the product purity **YES** **NO**

Improving the efficiency/lowering cost **YES** **NO**

Making it more environmental friendly **YES** **NO**

Please indicate your level of agreement with the following statements about the Reaction Optimization Assignment: (please circle one answer to each statement)

3. I clearly knew what was expected of me from the Assignment:

Strongly agree **Agree** **Neither Agree or Disagree** **Disagree** **Strongly disagree**

4. I felt like my optimization worked overall:

Strongly agree **Agree** **Neither Agree or Disagree** **Disagree** **Strongly disagree**

5. My/our team work was effective:

Strongly agree **Agree** **Neither Agree or Disagree** **Disagree** **Strongly disagree**

6. This Assignment prepared me for the Synthesis Assignment:

Strongly agree **Agree** **Neither Agree or Disagree** **Disagree** **Strongly disagree**

7. This Assignment was helpful in improving my laboratory and critical thinking skills:

Strongly agree **Agree** **Neither Agree or Disagree** **Disagree** **Strongly disagree**

8. This Assignment helped me to become more aware of industrial processes: *(if I were to work as a chemist in industrial settings this knowledge would help me become more proactive in the optimization of my assigned work):*

Strongly agree Agree Neither Agree or Disagree Disagree Strongly disagree

9. This Assignment is essential for a student pursuing a degree in chemistry

Strongly agree Agree Neither Agree or Disagree Disagree Strongly disagree

10. This experiment was useful preparation for seeking/obtaining a job as a chemistry laboratory technician in an academic or industrial setting.

Strongly agree Agree Neither Agree or Disagree Disagree Strongly disagree

11. <u>Specifically regarding this assignment</u> , can you suggest anything that the instructor might do differently in the future that will enhance the analytical and critical thinking skills of student participants?

12. Is there anything else you would like to tell us about this assignment?

Thank you for taking the time to share your feedback. It is much appreciated.

Survey Results on the Impact of Reaction Optimization Experiment

Student survey was given at the end of each of the 4 semesters of implementation. The survey used a Likert scale questions for assessing the learning outcomes for this project and included two free response questions for suggestions. 33 students were surveyed. Disruptions due to COVID resulted in the experiment not implemented in Spring 2020. The results of the survey are given below (tables 1, and 2. Question 1 in table 1 provided us information on the prior knowledge on SciFinder software use. SciFinder, a product of Chemical Abstracts Service (CAS), is a comprehensive literature search tool for chemical literature. Knowing how to search the chemical database by chemical structures and chemical reactions is a critical skill set for chemistry students. 23 out of 33 students had not used this tool prior to their capstone course.

Question 2 looked at the student perspectives on the outcome of their chosen reaction modification towards the goal of reaction optimization. The success in optimization was measured using four parameters. (a) An increase in product yield, which was achieved by 19 students. (b) Improving product purity, reported by 15 students. (c) Improving the efficiency in terms of economy of the reaction, 10 students felt they achieved this goal. (d) Making the reaction more environment friendly by using green reagents, 16 students felt they have been able to switch to green reagents through their modifications.

Table S1: Survey with Likert responses on knowledge of literature search platform and success in achievement of the goals of their reaction modifications

**Question**	**Description**	**Yes**	**No**
1	Did you know about the SciFinder tool before this class?	10	23
2	Did your reaction Optimization work in terms of		
	(a) Increasing the product yield	19	14
	(b) Improving the product purity	15	18
	(c) Improving the efficiency/lowering cost	10	23
	(d) Making it more environmentally friendly	16	17

The next set of questions in the survey focused on their personal opinion on the process and the impact of this experience on the development of their technical and soft skills. Majority of the students agreed on the expectation for the project, success of their optimization method and that their teamwork was effective. The major goals of the project that included development of soft skills, incorporating CURES in the project, critical thinking, and preparing them for employment or a graduate school research work were achieved as per the feedback from the students in the survey (table 2, questions 4-8).

Table S2: Survey with Likert's responses on the goals of the project

Question	Description	Strongly agree	Agree	Neither agree or disagree	Disagree	Strongly disagree
1	I clearly knew what was expected of me from the assignment	9	15	7	2	0
2	I felt like my optimization worked overall	4	16	8	2	3
3	My/our team work was effective	12	17	2	2	0
4	This assignment prepared me for the synthesis assignment	14	15	4	0	0
5	This assignment was helpful in improving my laboratory and critical thinking skills	20	13	0	0	0
6	This assignment helped me to become more aware of industrial processes (If I were to work as a chemistry in industrial settings this knowledge would help me become more proactive in the optimization of my assigned work)	14	15	4	0	0
7	This assignment is essential for a student pursuing a degree in chemistry	14	13	6	0	0
8	This assignment was useful preparation for seeking/obtaining a job as a chemistry laboratory technician in an academic or industrial setting	16	12	3	0	0

The final two questions on the survey were free response questions where the students provided suggestions on improving this project. Several students wanted more instructions that are detailed and examples on the types of modifications that can be performed to a reaction. The initial handout given to the students will be supplemented with examples of reaction modifications in the future. 29 students thought the experiment both prepared them for organic synthesis and made them more aware of the industrial processes (4 students were neutral). 29 students thought that their teamwork was effective, 2 students were neutral and 2 disagreed. 27 students thought that this experiment was essential for the students pursuing a degree in

chemistry (6 were neutral); and 28 students agreed, of whom 16 strongly agreed, that this assignment was useful in preparation for seeking a chemistry laboratory technician job (3 were neutral).

Grading Rubric for Oral Presentation:

1. Organization/Professional Demeanor: Did your talk clearly show how you performed your project? Do you have a good introduction? Do you state the significance of your project? Do you clearly state your accomplishments and conclusions? (5 points)

2. Clarity: Were your visual aides effective in helping the audience understand your project? Did you talk clearly and in an understandable manner? (3 points)

3. Content: Was your experimental data complete? Was the chemistry of your project fully explained? Did you show mechanism with correct arrows, name, and explain it? (8 points)

4. Analysis: Was your conclusion supported by the experimental results? Was your spectral data discussed in a manner complete enough for the audience to understand that you accomplished your goal? Did you clearly explain your successes and give rationales for any failures? What would you do differently? Did you answer questions from the audience and the instructor? (6 points)

5. Participation in discussion of your peers' reports. Have you posted at least one meaningful question to your peers? (3 points)
